# Development and Validation of an Index Based on EAT-Lancet Recommendations: The Planetary Health Diet Index

**DOI:** 10.3390/nu13051698

**Published:** 2021-05-17

**Authors:** Leandro Teixeira Cacau, Eduardo De Carli, Aline Martins de Carvalho, Paulo Andrade Lotufo, Luis A. Moreno, Isabela Martins Bensenor, Dirce Maria Marchioni

**Affiliations:** 1 Department of Nutrition, School of Public Health, University of São Paulo, São Paulo 01246-904, Brazil; lcacau@usp.br (L.T.C.); edecarli@usp.br (E.D.C.); alinenutri@usp.br (A.M.d.C.); 2 Clinical and Epidemiological Research Center, University Hospital, University of São Paulo, São Paulo 05508-000, Brazil; palotufo@usp.br (P.A.L.); isabensenor@gmail.com (I.M.B.); 3 Growth, Exercise, Nutrition and Development (GENUD) Research Group, Faculty of Health Sciences, University of Zaragoza, 50009 Zaragoza, Spain; lmoreno@unizar.es; 4 Instituto Agroalimentario de Aragón (IA2), Instituto de Investigación Sanitaria de Aragón, 50013 Zaragoza, Spain; 5 Centro de Investigación Biomédica en Red de Fisiopatología de la Obesidad y Nutrición (CIBEROBN), Instituto de Salud Carlos III, 28040 Madrid, Spain

**Keywords:** diet quality, sustainable diet, diet indexes, EAT-Lancet diet

## Abstract

The EAT-Lancet Commission has proposed a planetary health diet. We propose the development of the Planetary Health Diet Index (PHDI) based on this proposed reference diet. We used baseline dietary data obtained through a 114-item FFQ from 14,779 participants of the Longitudinal Study on Adult Health, a multicenter cohort study conducted in Brazil. The PHDI has 16 components and a score from 0 to 150 points. Validation and reliability analyses were performed, including principal component analyses, association with selected nutrients, differences in means between groups (for example, smokers vs. non-smokers), correlations between components and total energy intake, Cronbach’s alpha, item-item correlations, and linear regression analysis between PHDI with carbon footprint and overall dietary quality. The mean PHDI was 60.4 (95% CI 60.2:60.5). The PHDI had six dimensions, was associated in an expected direction with the selected nutrients and was significantly (*p* < 0.001) lower in smokers (59.0) than in non-smokers (60.6). Cronbach’s alpha value was 0.51. All correlations between components were low, as well as between components and PHDI with total energy intake. After adjustment for age and sex, the PHDI score remained associated (*p* < 0.001) with a higher overall dietary quality and lower carbon footprint. Thus, we confirmed the PHDI validity and reliability.

## 1. Introduction

The human diet has been transformed in recent decades due to technological advances, globalization, and changes in agricultural systems [1]. In addition to these changes, the definition of a healthy diet has been discussed and remodeled, with the aim of including planetary health concepts [2].

According to the Food and Agriculture Organization (FAO) and World Health Organization (WHO), sustainable healthy diets are “dietary patterns that promote all dimensions of individuals’ health and wellbeing; have low environmental pressure and impact; are accessible, affordable, safe and equitable; and are culturally acceptable [3]”.

In relation to this, the EAT-Lancet Commission on “Healthy Diets from Sustainable Food Systems” (EAT-Lancet) proposed a healthy and sustainable model diet that aims to provide health to the population and the planet, called the “Planetary Health Diet.” These recommendations are based on predominant consumption of vegetables, greens, fruits, and whole grains, and reduced consumption of meat, fish, eggs, refined cereals, and tubers [4].

This diet model has been debated in the scientific community regarding to its cost and affordability [5,6], compared with guidelines for healthy dietary patterns [7] and the Indian diet [8], and even used as a reference for a novel Danish plant-based diet [9], besides being widely disseminated in several languages (EAT-Lancet Commission Summary Report: https://eatforum.org/eat-lancet-commission/eat-lancet-commission-summary-report/ accessed on 23 April 2021). In addition, two indices for assessing adherence to the EAT-Lancet reference diet have been proposed [10,11]; however, they have some limitations. While one of them uses a binary scoring criterion [10], the other uses a gradual scoring [11]; nevertheless, both use reference values in grams, which does not allow for the assessment of individuals’ adherence regardless of the energy content of the diet. In addition, they do not include all intermediate values and interchangeable groups, as proposed in the EAT-Lancet report itself. Thus, it would be advantageous to have an index that considers these characteristics, in addition to using a density base to enable the evaluation of different caloric scenarios. Other than that, the EAT-Lancet reference diet must be both healthy and sustainable. Therefore, an index that has its performance tested in relation to measures of diet quality and environmental impact is missing. In this way, in this article, we propose the development and validation of the Planetary Health Diet Index (PHDI) using data from the Longitudinal Study on Adult Health (ELSA-Brasil), a well-developed cohort study in Brazil.

## 2. Materials and Methods

### 2.1. Study Population

This cross-sectional study used the baseline data from ELSA-Brasil, a multicenter cohort of 15,105 men and women aged 35–74 years, who were active and retired workers from six public universities from three major Brazilian regions (Northeast, Southeast, and South). Baseline data from ELSA-Brasil were collected between August 2008 and December 2010. Details of the sample and data collection methods of this study were previously published [12,13,14]. Briefly, baseline measures included anthropometry (height and body weight), food consumption using a semi-quantitative Food Frequency Questionnaire (FFQ), and sociodemographic and lifestyle data using a general questionnaire (sex, age, smoking status, and physical activity level at leisure time using the International Physical Activity Questionnaire (IPAQ)). For the present analysis, we disregarded participants without food consumption information (*n* = 24) and those who were below the 1st and above the 99th percentile of dietary energy intake (*n* = 302), in order to exclude possibly invalid food intake data. The final sample for analysis included 14,779 individuals.

The ELSA-Brasil was approved by the research ethics committees of all research centers. All participants volunteered and signed an informed consent form. The present study was also approved by the research ethics committee of the School of Public Health of the University of São Paulo (number 3.970.703).

### 2.2. Dietary Assessment

The usual dietary intake of participants in the last 12 months was assessed using a 114-item semi-quantitative FFQ, previously validated for ELSA-Brasil [15,16]. Participants had to report their consumption frequency (more than 3 times/day, 2–3 times/day, once a day, 5–6 times a week, 2–4 times a week, once a week, 1–3 times a month, and never/almost never) and the quantity consumed using standard portion sizes. The daily consumption of each FFQ item (in g/day) was obtained by multiplying the portion size by the corresponding frequency. Food measurements were then converted into nutrient intakes using the United States Department of Agriculture (USDA) Food Composition Database, except when its values were outside the range of 80% to 120% from those described in the Brazilian Table of Food Composition, where the latter reference was used [17,18].

### 2.3. Development of the Planetary Health Diet Index (PHDI)

The Planetary Health Diet Index (PHDI) was adapted from the recommendations of the reference diet proposed by the EAT-Lancet Commission [4]. Briefly, this reference diet was set as a daily intake of 2500 kcal with possible ranges of contributions from 23 different food groups expressed as both g/day and kcal/day. To adapt these recommendations in a manner that easily accommodates different caloric scenarios, all ranges and midpoints proposed for each food group were calculated as their energetic contribution to the reference diet of 2500 kcal/d. The PHDI components and their cutoff and thresholds were then defined from these values, taking into account the exchangeability and interchangeability of some food groups, as detailed in Table 1.

For each of the 16 components set in the PHDI, a maximum of 10 or 5 points could be ascribed, resulting in a total score ranging from 0 to 150 points. As illustrated in Table 1, components were categorized into four different scoring classifications: adequacy, optimum, ratio, and moderation, depending on how their intake values indicate higher or lower adherence to the reference diet assumptions, following a system adapted from Looman et al. [19].

Food groups stated as adequacy components were those for which one could assume that intakes equal to zero (i.e., non-consumption) have a lower dietary quality, while intakes equal to or above the reference value would not likely imply high detriments to human and planetary health. Thus, nuts and peanuts, legumes, fruits, total vegetables, and whole grains were defined as adequacy components. Similar to the adequacy components, the optimum components were selected as food groups for which a certain minimum level of intake (that is, the midpoint values) would be preferred over non-consumption. However, consumption that approaches or exceeds an upper limit, based on the maximum consumption values recommended in the reference diet, could gradually decrease the sustainability and diet quality. Eggs, fish and seafood, tubers and potatoes, dairy, and unsaturated oils were defined as optimum components.

The two ratio components in the PHDI are indicative of a compositional distribution of dark green vegetables and red and orange vegetables in relation to the total vegetable intake. Thus, to avoid an overrating of that specific dietary dimension under evaluation, we decided to assume a maximum score of only 5 points for each ratio component. In opposition to the adequacy components, food groups selected as moderation components were assumed to imply a higher quality and sustainability of diets when intakes are equal to or approaching zero (cutoff). Red meat, chicken and substitutes, animal fats, and added sugars were defined as moderation components in PHDI. More details on each component are described below.

- Nuts and peanuts: This component was defined by the combination of the proposed mean intake points for peanuts (5.7%) and nuts (5.9%) in the reference diet as a single cutoff value of 11.6% of the total calories. Thus, the recommendation is an intake of 11.6% of total calories, then an intake equal to or higher than that was scored as 10 points. An intake equal to 0% was scored as 0 points, while an intake from 0% to 11.6% was proportionally scored between 0 and 10 points. Processed and raw nuts, pistachios, almonds, peanuts, and coconut pulp and milk were included in this PHDI component.
- Legumes: In the reference diet, plant protein sources proposed as interchangeable, such as legumes (including soy), nuts, and peanuts, have a very large allowance for their total intake (up to 39.7% of total calories) [4]. At least in a traditional Western-style dietary pattern, it is uncommon for individuals or combined intake of these plant protein sources to exceed such a high-shared limit. Therefore, we set legumes as an adequacy component in the PHDI, summing the midpoint intakes proposed for beans, lentils, and peas (6.9%) and for soy foods (4.5%) to define a single cutoff value of 11.4% of the total calories. Thus, an intake equal to or higher than that was scored as 10 points. An intake equal to 0% was scored as 0 points and an intake from 0% to 11.4% was proportionally scored between 0 and 10. This food group included all dry or canned beans, pulses, lentils, chickpeas, peas, soybeans, and soy food products.
- Fruits: Fruits had minimum and maximum intake limits proposed in the reference diet, despite the benefits recognized in high intakes [4]. In this sense, we take fruit as a component of PHDI adequacy and assign a maximum score (10 points) to all intakes that exceed the cutoff point of 5.0% of total calories. Intake of 0% was scored with 0 points, while consumption from 0% to 5.0% was gradually scored from 0 to 10 points. All fresh and processed fruits were considered, including its fractioning contribution in culinary or industrial products such as fruit juices, nectars, and punches. Due its particular nutrient composition profile and pattern of consumption, coconut water was also counted as a fruit.
- Total vegetables: This adequacy component, similar to what was argued for fruits, had the maximum score (10 points) for all intakes that exceed the cutoff point of 3.1% of total calories. An intake of 0% was scored with 0 points, while consumption from 0% to 3.1% was scored gradually from 0 to 10 points. Items accounting for this food group included fresh, frozen, cooked, canned, or dried vegetables and excluded legumes and starchy vegetables.
- Whole grains: In the reference diet, no specific maximum consumption limit was proposed for whole grains [4], despite the desirable limitation of diets to less than 60% of the energy contribution of carbohydrates. Therefore, the intake of 32.4% of the total calories of the reference diet was defined as the cutoff point in the PHDI. Thus, the intake of 32.4% or higher was scored with 10 points. An intake of 0% was scored as 0 points, while an intake from 0% to 32.4% was scored proportionally between 0 and 10. The foods included in this component are whole grains used as staple foods (for example, brown rice, brown bread, oat flakes, canned grains, etc.), but not processed grains or refined product flours, such as polished rice and white bread, cookies, pasta, sweets, and breakfast cereals.
- Eggs: This group has an average intake point of 0.8% of the total calories of eggs in the reference diet, with an upper limit of up to 1.5% of the total calories, considering its potential benefit for the quality of the diet in low-income populations [4]. Using these values as the cutoff point and upper limit of the PHDI, respectively, an intake from 0% to 0.8% was scored gradually from 0 to 10 points, while consumption from 0.8% to 1.5% was scored inversely. An intake above 1.5% received 0 points. This food group includes eggs from chickens and other poultry.
- Fish and seafood: Fish intake brings benefits to human health, especially related to its important contribution to the essential omega-3 fatty acids in the diet and can serve as an alternative to direct the production of animal proteins for lower environmental impacts [4]. In the reference diet, the intake of this group had an average point of 1.6% and an upper limit of 5.7% of total calories. Thus, an intake from 0% to 1.6% was scored gradually from 0 to 10 points, while an intake from 1.6% to 5.7% was scored inversely. An intake above 5.7% was scored as 0. This group includes fish and seafood.
- Tubers and potatoes: Although they are an important staple food for many populations, potatoes, cassava, and their derivatives generally provide high glycemic loads, which can increase the risk of weight gain, diabetes, and cardiovascular diseases [4]. Thus, the midpoint value of 1.6% of total calories was assumed to be an optimal intake for this group. However, the cutoff point of 3.1% of total calories was considered an upper limit in PHDI. Thus, the intake from 0% to 1.6% was scored gradually from 0 to 10 points. An intake from 1.6% to 3.1% was scored inversely, while the intake above 3.1% was scored at 0 points. This group included all types of potatoes and cassava, as well as their derivative products.
- Dairy: To guarantee approximately 718 mg/day of calcium in the reference diet, an average point intake of 6.1% of total calories was defined by the EAT-Lancet Commission [4]. The target value was assumed to be the ideal intake for this group in PHDI. In contrast, an intake of approximately double that, which is 12.2% of total calories, was defined as the upper limit value for dairy foods. Thus, an intake from 0% to 6.1% was scored gradually from 0 to 10 points, while an intake from 6.1% to 12.2% was scored inversely. Intakes above the maximum limit were scored as 0. This group includes cow’s milk, goat’s milk, buffalo products, yogurt, and cheese, but no butter and sour cream.
- Vegetable oils: Vegetable oils were allowed with a wide range of consumptions in the reference diet; however, the EAT-Lancet Commission made a distinction of recommendations for specific palm oil, given its undesirable fatty acid profile [4]. In addition to its culinary application in some typical dishes from certain regions of Brazil and Africa, palm oil is mainly an ingredient in the food industry that is difficult to quantify separately from other vegetable oils using common dietary assessment methods and databases of available food composition. Therefore, we decided to set a single limit of 16.5% of total calories for a broad component of vegetable oil, combining the proposed midpoint intakes for palm oil (2.4%) and vegetable oils (14.1%) in the reference diet. A similar addition was also considered in the upper reference limit of vegetable oil intake to define the limit of 30.7% of the total calories for the vegetable oil component. Thus, an intake from 0% to 16.5% was scored gradually from 0 to 10 points, while an intake from 16.5% to 30.7% was scored inversely. Ingestion above the upper limit received 0 points.
- Dark green vegetables to total vegetables ratio: The reference diet emphasizes that most of the health benefits of vegetables are likely to be achieved by diversifying their total energy contribution [4]. Therefore, we defined the value of 29.5% as the cutoff point for the present component, calculated as the ratio between the energy intake of dark green vegetables (numerator) and the total of vegetables (denominator) multiplied by 10. Values from 0% to 29.5% were scored gradually from 0 to 5 points, while values above the cutoff point were scored inversely. All dark green vegetables were included, such as broccoli and rocket, but not light green vegetables, such as lettuce.
- Red and orange to total vegetables ratio: The limit value of 38.5% was defined to assess the ratio between the energy intake of the red and orange vegetables (numerator) and the total of vegetables (denominator) multiplied by 10. Thus, values from 0% to 38.5% were scored gradually from 0 to 5 points, while values above the cutoff point were scored inversely. Tomatoes, beets, carrots, and pumpkins are examples of foods included in this component.
- Red meats: In the reference diet, an exchangeable range of tolerable intakes is proposed for beef, lamb, and pork, despite being considered not essential and with a target contribution of zero calories daily [4]. Taking this into account, we set red meat as a moderation component in the PHDI, combining the upper boundary intakes proposed for beef and lamb (1.2%) and pork (1.2%) to define a single threshold value of 2.4% of the total calories. Thus, an intake above this upper limit scored 0 points, while zero intakes were awarded a maximum of 10 points. An intake from 0% to 2.4% was scored inversely and gradually from 0 to 10 points. Processed beef and pork (for example, sausages, ham, bologna, dried meat) were also accounted for along with cuts of fresh meat in this component.
- Chicken and substitutes: This group of chickens and other poultry should have an ideal consumption of zero to approximately 58 g/day, even if it can be exchanged for eggs, fish, or plant protein sources [4]. According to the EAT-Lancet, chicken and other poultry can be exchangeable with eggs, fish and seafood, and plant protein sources [4]. In order to consider this characteristic, we assumed that any percentage of the energy intake of eggs and/or fish and seafood that eventually exceeds their respective recommendation limits could be accommodated as part of the proposed intake range for chickens and other poultry. We did not consider any substitution of plant protein sources, considering its frequent consumption from low to moderate by the populations considered. Therefore, for chickens, poultry, and their substitutes (i.e., eggs and fish and seafood), a single intake limit was set using the upper limit of 5.0% of the total calories in the reference diet. Processed poultry meat (for example, smoked brisket and nuggets) was also accounted for along with cuts of fresh meat in this component. Thus, an intake above this upper limit scored 0 points, while zero intakes were awarded a maximum of 10 points. An intake from 0% to 5.0% was scored inversely and gradually from 0 to 10 points. Processed meat from poultry (e.g., smoked breast, nuggets, and pate) were also accounted for along with fresh meat cuts in this component.
- Animal fats: As an optional allowance in instances when pigs or cattle are consumed, an intake of up to 5 g daily of lard and tallow is tolerated in the reference diet. In turn, dairy fats have a target contribution of zero intakes [4]. Despite any reference to the interchangeability of these components by the EAT-Lancet Commission, we decided to merge dairy fats, lard, and tallow food groups, assuming a threshold of 1.4% of total calories as the maximum tolerance for animal fat intake. Thus, an intake above this upper limit scored 0 points, while zero intakes were awarded a maximum of 10 points. An intake from 0 %to 1.4% was scored inversely and gradually from 0 to 10 points. Butter and creams (e.g., sour cream and cheese cream) were counted along with lard and tallow in this moderation component.
- Added sugars: The threshold of 4.8% of total calories from the reference diet is used in the PHDI to assess and score all sweetener intake, including the table white or brown sugars and honey used as ingredients in processed or culinary products and the added sugars to manufactured foods and beverages. Similar to all other moderation components, zero intakes were awarded a maximum of 10 points. Thus, an intake above this upper limit scored 0 points, while zero intakes were awarded a maximum of 10 points. An intake from 0% to 4.8% was scored inversely and gradually from 0 to 10 points.

### 2.4. Extracting PHDI Components from Food Consumption Data

To calculate energy intake values from single food groups, as required for PHDI component computations, all mixed dishes and processed products from the FFQ (e.g., pan fried vegetables, pizza, soups, cakes, etc.) were decomposed into individual ingredients based on household standard recipes from the national literature [20,21]. Household standard recipes were also used for ingredients decomposition of manufactured foods (e.g., plain cookies, fruit nectars, granola, etc.). In particular, for highly processed products based on one major component (e.g., savory chips based on maize starch, filled cookies based on wheat flour, and instant noodles based on wheat flour), we estimated the fractioning of total energy across its ingredients on the basis of the contents of added sugars and total fat in the food, as described in the nutrient database. For instance, the energetic percentage of total fats in a savory chip was considered to be the fraction of the vegetable oil group’s contribution to that food (vegetable oils and/or palm oil). Meanwhile, the remaining energetic value after discounting the total fats from the savory chips was assumed to be the refined cereals group’s contribution to that food (maize starch). Exceptionality, processed meats were not decomposed into ingredients, but classified according to their predominant ingredient origin or most commonly marketed formulation into the respective red meat (e.g., sausage, ham, and salami) or chicken and substitutes (e.g., pate, nuggets, etc.) groups. To calculate the participants’ total energy and each component intake (expressed as percentages of total energy intake or as ratios between two percentages of total energy intake), the contribution of all consumed foods was considered, except alcoholic beverages, given their non-inclusion in the reference diet [4]. Table S1 shows examples of foods and ingredients included in the PHDI components.

### 2.5. Validity and Reliability of the Planetary Health Diet Index (PHDI) 

The performance of the PHDI was measured using strategies for assessing construct validity and reliability, as proposed by Reedy et al. [22]. In addition, we checked the validity of the PHDI by relating it to overall dietary quality evaluated by a national revised tool [23] and with an environmental impact measure assessed through the dietary carbon footprint estimation [24].

#### 2.5.1. Construct Validity 

Construct validity evaluates how well an index measures what it should measure [25]. Thus, in order to verify the construct validity of PHDI, we evaluated some criteria. First, we assessed the correlation between the total PHDI score with selected nutrients, in order to assess if the correlations went in an expected way (for example: does animal protein source consumption decrease as the PHDI score increases?). For that, we used linear regression models adjusted for sex and age.

Second, we investigated whether PHDI can assess adherence to EAT-Lancet recommendations regardless of the amount consumed in the diet. For this, we tested the correlations between the total score and its components with the total energy intake. To assess this independence, we examined Pearson’s correlations of total score and PHDI components with total energy intake [25].

Third, we investigated whether PHDI had more than one factor that explained the variability of the data. For that, we used the principal component analysis (PCA) to access the correlation between the 16 components to check if there was more than one factor that explains the variability. In the PCA analysis, the correlation matrix was obtained using varimax rotation and only eigenvalues > 1 was used to determine the number of factors [26]. The Scree test was used as an auxiliary method, as it allowed us to present the amount of variation of each of the main components or factors [27].

#### 2.5.2. Concurrent-Criterion Validity

To evaluate whether the index can distinguish between groups with known differences in the quality of their diets [25], PHDI was compared in terms of average scores between sex, smokers and non-smokers, adults and the elderly, and between physical activity levels. For discrimination analysis, comparisons between the groups were performed using a Student’s t-test or ANOVA.

#### 2.5.3. Internal Reliability

Internal reliability measures the degree to which various components of an index measure the same construct [25]. Thus, we use Cronbach’s alpha to assess whether the components of PHDI measured the same construct. This statistic assesses the average of the correlations between all possible combinations, in this case, of the 16 components of PHDI [25]. We also evaluated the item-item correlation between the components to better understand the relationships between them.

#### 2.5.4. Overall Dietary Quality and Dietary Carbon Footprints 

The overall dietary quality was assessed using the Brazilian Healthy Eating Index-Revised (BHEI-R) [23,28]. This index is composed of 12 components: 9 food groups (total fruit, whole fruit, total vegetables, dark green and orange vegetables, total grains, whole grains, milk and dairy, meat, eggs and legumes, and oils), 2 nutrients (saturated fat and sodium), and the sum of energy from solid fat, alcohol, and added sugar (the SoFAAS component). The BHEI-R can range from 0 to 100 and is estimated per 1000 kcal [23]. More information can be found in the study of Previdelli et al. [23].

We used the “Environmental Footprints of Food and Culinary Preparations Consumed in Brazil”, a database built from a literature review of food life cycle assessments (LCA), to estimate the carbon footprint from the individual consumption data assessed with the FFQ. More information about this database can be found elsewhere [24]. Briefly, we estimated the average greenhouse gas emissions (GHGE) per person per day through the sum of CO_2_eq from all food consumed by individuals. After that, we adjusted the GHGE by total energy intake, dividing the CO_2_eq from diet per calorie intake and multiplying by 1000. The relationship between the PHDI and the BHEI-R and between the PHDI and the carbon footprint was explored using linear regression models adjusted for sex and age.

### 2.6. Statistical Analysis

All analyses were performed using Stata statistical software (release 14, 2015, StataCorp LP, College Station, TX, USA), and the level of significance was set at 5%.

## 3. Results

The PHDI presented a normal distribution (Figure S1) and a mean score of 60.4 (95% confidence interval (95% CI) 60.2:60.5). As shown in Table 2, some components had higher average scores, such as fruits, vegetables, chickens and substitutes, and legumes, while the red meat, whole cereals, and nuts and peanuts components had lower average scores.

### 3.1. Construct Validy

The total PHDI score was associated in an expected direction with the selected nutrients, showing a positive association (*p* < 0.001) with carbohydrates, vegetable proteins, polyunsaturated fats, fibers, vitamins A, E, K, C, thiamine, folate, iron, phosphorus, potassium, zinc, selenium, magnesium, and copper (*p* = 0.029), and negatively associated (*p* < 0.001) with animal protein, total fat, saturated fat, cholesterol, monounsaturated fat, riboflavin, niacin, vitamin B5, pyridoxine, and vitamin B12. The index was not associated with total energy, total protein, calcium, or sodium (Table 3).

The correlations between each PHDI component and the total energy were all low. The highest absolute correlations were between energy and beans and soy (0.23), the red and orange vegetables ratio (−0.21), and the dark green vegetables ratio (−0.20). The correlation between the total energy and total score was also low (−0.02) (Table S2).

**Figure 1:**
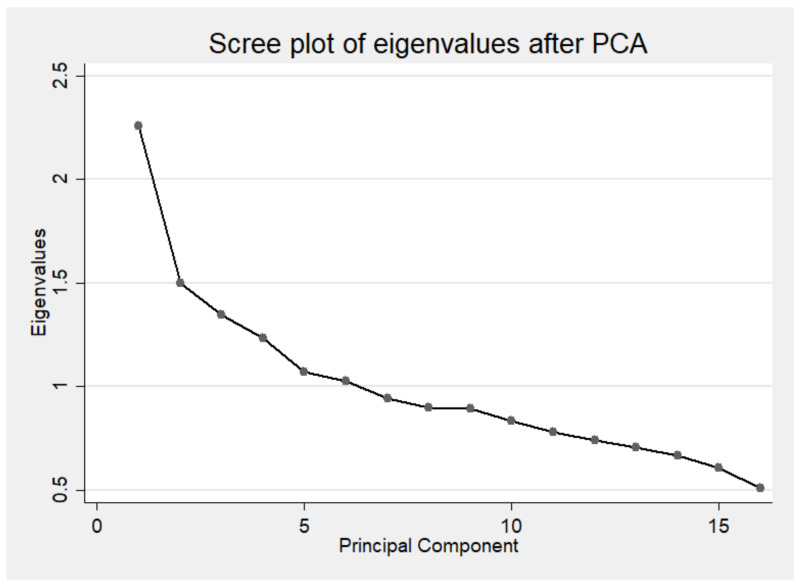
Scree plot from principal components analysis (PCA) of Planetary Health Diet Index. ELSA-Brasil, 2008–2009.

The PCA revealed that several factors explain the PHDI variability. The Scree plot illustrated that no single linear combination of the 16 components of PHDI was responsible for a significant proportion of the covariance of the data. Figure 1 shows the presence of six factors with an eigenvalue > 1 and that the line seems to stagnate after the seventh factor.

### 3.2. Concurrent-Criterion Validity

Regarding the difference between groups, non-smokers, the elderly, and those with a moderate and vigorous level of physical activity had higher average scores when compared to smokers, adults, and those with a low level of physical activity (Table 4).

### 3.3. Internal Reliability

Cronbach’s alpha value was 0.51. The higher correlations were found between the components of ReV/total ratio and tubers (0.45), between the DGV/total ratio and tubers (0.32), and between animal fat and added sugars (0.27). In general, all correlations between the components were low to moderate, varying between 0.01 and 0.45 (Table S2).

### 3.4. Overall Dietary Quality and Dietary Carbon Footprints

The BHEI-R average was 70.3 (95% CI, 70.2:70.5) and the carbon footprint was 1.93 kg/COeq/1000kcal (95% CI, 1.92:1.94). Women and the elderly had higher diet quality scores. Regarding the carbon footprint, women had a higher average GHGE emission (Table S3).

The PHDI score was significantly associated with the overall quality of the diet, assessed by BHEI-R and the carbon footprint. After adjusting for age and sex, the PHDI score remained positively associated with the overall quality of the diet, indicating that those with higher PHDI scores have higher diet quality scores. Other than that, PHDI was negatively associated with the carbon footprint, indicating that those with higher PHDI scores have lower GHGE emissions (Table 5).

## 4. Discussion

In this study, we developed and validated an indicator for assessing adherence to the recommendations of the healthy and sustainable diet proposed by the EAT-Lancet Commission. We observed that PHDI performed satisfactorily in terms of validity and reliability and was associated with higher overall dietary quality and lower carbon footprint. 

Construct validity was confirmed according to the established criteria, such as being associated with selected nutrients in expected directions. The final score was negatively associated with animal protein, total fat, saturated fat, cholesterol, and monounsaturated fat. These results were already expected, as individuals who had excessive consumption of the food group sources of these nutrients would receive a score of zero, as their consumption is associated with adverse health and environmental effects [4]. In addition, PHDI was negatively associated with the micronutrients present in these food groups. In contrast, PHDI was positively associated with carbohydrate, vegetable protein, polyunsaturated fat, fiber, and micronutrients present in fruits, vegetables, oilseeds, and whole grains [4]. 

In addition, construct validity was demonstrated with the low correlations between the total score and its components with the total energy [25]. The correlation between the final score and energy was -0.02, indicating that the index evaluates adherence to the EAT-Lancet diet regardless of the calorie consumption, since it scores according to the caloric densities of the reference diet. The PCA presented six factors, indicating that no linear combination of the 16 components is responsible for explaining a substantial amount of the variation in the index. Construct validity was also confirmed by the comparison of the final score between groups with established differences in dietary quality, demonstrating that non-smokers, the elderly, and individuals with moderate to vigorous physical activity level have higher adherence to EAT-Lancet diet when compared to smokers, adults, and individuals with low levels of physical activity (i.e., sedentary), respectively. These results demonstrate good PHDI construct validity, as demonstrated in other studies on the development and validation of diet assessment indexes [22,25,29,30]. 

Cronbach’s alpha was 0.51. Although values ≥ 0.6 are considered desirable, some authors found values from 0.22 to 0.68 in different indexes of dietary evaluation [25,31,32,33,34]. It is necessary to consider that the Cronbach’s alpha can be influenced if the construct is one-dimensional or multidimensional and if the items are homogeneous with each other [25], in addition to being affected by sample characteristics, such as being heterogeneous or homogeneous [25]. As described, the PHDI proved to be multidimensional, in addition to being applied in a large population [12]. Thus, this result is consistent, since the diet is known to be a complex and multidimensional construct [25]. In this way, the Cronbach’s alpha value found with the PHDI can be considered acceptable, as it was expected that this coefficient would be lower due to the characteristics mentioned above.

Finally, the final PHDI score was positively associated with the overall dietary quality assessed by the BHEI-R, demonstrating that individuals with better dietary quality have higher adherence to the EAT-Lancet reference diet. This result indicates that PHDI can capture the quality of an individual’s diet. In addition, PHDI was also associated with a lower carbon footprint, indicating that the lower the GHGE emission, the higher the PHDI score. These results were expected, because according to the report, the reference diet is qualitative and nutritionally adequate and has low environmental impact [4]. Thus, according to the best of our knowledge, this is the first study to present the development and validation of an index based on the recommendations of the EAT-Lancet, including measures of food quality and environmental impact, which includes the intermediate values and interchangeable groups as proposed in the EAT-Lancet report itself.

Regarding the two indices developed based on the recommendations of the EAT-Lancet, it is important to highlight that one of them, the EAT-Lancet diet score, is a binary score index ranging from 0 to 14 points that was developed and applied to participants in the EPIC-Oxford study and considered to be consumption below the minimum recommendation or consumption above the maximum recommendation of certain food groups [10]. Despite being inversely associated with diabetes and ischemic heart disease [10], this index does not consider all the possibilities of the EAT-Lancet recommendations, such as the minimum and maximum values [35]. In addition, this index was negatively associated with micronutrients adequacy among women in five low- and middle-income countries (LMCC) [36]. The PHDI has a gradual score, which allows for a better distinction between the degrees of adherence of individuals, favoring an interpersonal distribution. In addition, it allows for a more refined association with the diet quality and the environment.

Another index based on the EAT-Lancet recommendations, the World Index for Sustainability and Health (WISH) proposed by Trijsburg et al., has 13 items ranging from 0 to 130 points, in addition to using a gradual score and recommendation values in grams [11]. Thus, the PHDI is similar to the WISH in the gradual scoring criterion, but it has some different characteristics that deserve to be highlighted. PHDI evaluates the calorie density recommendations of the reference diet to allow for the assessment of different calorie scenarios, which allows for assessing adherence regardless of the caloric amount of the diet. In addition, the PHDI considers tubers and groups of interchangeable foods, such as eggs and fish with chicken and other poultry, making the PHDI more similar to the recommendations of the reference diet [4].

Developing a dietary index can be complex and challenging as it involves a series of subjectivities related to its construction, its components, cutoff points, and the score scale used [37,38]. Therefore, PHDI followed some recommendations to become a good performance index, such as using the gradual scoring criterion [38] and considering all the recommended values of the reference diet [4]. As a result, we obtained an index that presents itself as a good indicator of adherence to the reference diet proposed by the EAT-Lancet Commission, showing satisfactory results in relation to criteria of validity and reliability.

However, this study has some limitations. First, the PHDI was developed based on the values of food consumption obtained by an FFQ. Although the ELSA-Brasil FFQ has been previously validated and the FFQ is one of the most used methods in epidemiological studies in evaluating the relationship between diet and health outcomes, it remains a method that has some bias, such as the finitude of the list of food and being a self-report method, which may introduce some memory bias [39]. Second, the PHDI has not been evaluated for the validity of predictive criteria, such as the ability to predict death and/or illness. However, these analyses are planned and will be performed soon. 

The PHDI showed good reliability and proved to be valid for use as an instrument to assess the population’s adherence to the recommendations of a healthy and sustainable diet proposed by the EAT-Lancet Commission with the potential to be used in different populations, since the PHDI is not linked to food culture and current dietary patterns of consumption for a given population, as we did not make adjustments to the recommended dietary reference values. As described, the PHDI indicated that the recommended values for some components will require efforts from various stakeholders to be reached, such as red meats. The use of PHDI is expected to assist in generating results that support the EAT-Lancet proposals and public policy planning and guidelines on the benefits of healthy and sustainable diets, with an emphasis on the proposal by the EAT-Lancet Commission.

## Figures and Tables

**Table 1:** Planetary Health Diet Index components, standards for scoring (caloric densities), and corresponding point values.

Components	Scores (Points) ^1^				
	0	5	10	5	0
**Adequacy component**					
Nuts and peanuts	0.0 |	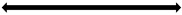	| ≥11.6		
Legumes ¢	0.0 |	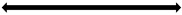	| ≥11.3		
Fruits	0.0 |	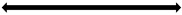	| ≥5.0		
Vegetables	0.0 |	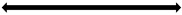	| ≥3.1		
Whole cereals	0.0 |	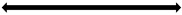	| ≥32.4		
**Optimum component**					
Eggs	0.0 |	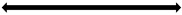	| 0.8 |	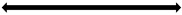	| ≥1.5
Fish and seafood	0.0 |	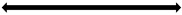	| 1.6 |	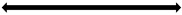	| ≥5.7
Tubers and potatoes	0.0 |	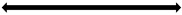	| 1.6 |	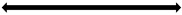	| ≥3.1
Dairy§	0.0 |	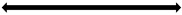	| 6.1 |	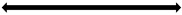	| ≥12.2
Vegetable oils º	0.0 |	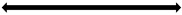	| 16.5 |	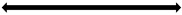	| ≥30.7
**Ratio component**					
DGV/total ratio ≠	0.0 | 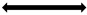 | 29.5			29.5 | 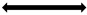	| 100
ReV/total ratio ≡	0.0 | 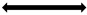 | 38.5			38.5 | 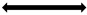	| 100
**Moderation component**					
Red meat £	≥2.4 |	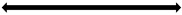	| 0.0		
Chicken and substitutes	≥5.0 |	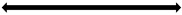	| 0.0		
Animal fats ǂ	≥1.4 |	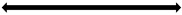	| 0.0		
Added sugars	≥4.8 |	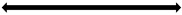	| 0.0		

^1^ All values expressed as caloric densities from the reference diet proposed by the EAT-Lancet Commission. The bars represent the limits. £ Red meat: beef, lamb, and pork. ¢ Legumes: beans and soy. § Dairy: excluding dairy fats. º Unsaturated oils: including palm oil. ≠ DGV/total ratio: ratio between the energy intake of dark green vegetables (numerator) and the total of vegetables (denominator) multiplied by 10. ≡ ReV/total ratio: ratio between the energy intake of red and orange vegetables (numerator) and the total of vegetables (denominator) multiplied by 10. ǂ Animal fat: lard, tallow, and dairy fats. DGV/total ratio: dark green vegetable/total ratio. ReV/total ratio: red vegetable/total ratio.

**Table 2:** Descriptive analysis of Planetary Health Diet Index components (values expressed as mean and standard deviation, and median and interquartile range). ELSA-Brasil, 2008–2009.

Components	Maximum Points	Mean	Standard Deviation	Median	IQR
Red meat	10	0.6	2.0	0	0–0
Nuts and peanuts	10	1.4	2.4	0.4	0–1.6
Legumes	10	5.2	3.2	4.8	2.3–8.1
Chicken and substitutes	10	6.4	2.9	7.2	4.8–8.6
Fish and seafood	10	4.5	3.6	5.1	0.0–7.9
Eggs	10	4.1	3.5	4.2	0.0–7.4
Fruits	10	9.7	1.2	10	10–10
Vegetables	10	9.9	0.7	10	10–10
DGV/total ratio	5	1.3	1.1	0.9	0.4–1.7
ReV/total ratio	5	1.7	1.1	1.4	0.9–2.2
Whole cereals	10	0.9	1.4	0.3	0–1.3
Tubers	10	1.8	3.0	0	0–2.9
Dairy	10	3.0	3.4	1.3	0–6.1
Unsaturated oils	10	3.9	1.6	3.7	2.7–4.9
Animal fats	10	3.8	3.8	3.1	0–7.3
Added sugars	10	2.3	3.0	0	0–4.5
Total score	0–150	60.4	11.5	60.0	52.2–67.9

DGV/total ratio: dark green vegetable/total ratio. ReV/total ratio: red vegetable/total ratio. IQR: interquartile range.

**Table 3:** Association between PHDI and nutrients. ELSA-Brasil, 2008–2009.

Nutrient	β ^a^	95% CI		*p*-Value
Energy (kcal)	−0.0002	−0.0005	−0.0001	0.125
Protein (g)	−0.0016	−0.0074	0.0041	0.577
Animal protein (g)	−0.1061	−0.1140	−0.0982	<0.001
Total fat (g)	−0.0377	−0.0452	−0.0302	<0.001
Cholesterol (mg)	−0.0339	−0.0355	−0.0323	<0.001
Saturated fat (g)	−0.2539	−0.2739	−0.2339	<0.001
Monounsaturated fat	−0.0739	−0.0970	−0.0508	<0.001
Riboflavin (mg)	−1.6658	−1.8967	−1.4349	<0.001
Niacin (mg)	−0.0456	−0.0580	−0.0328	<0.001
Vitamin B5 (mg)	−0.3490	−0.4285	−0.2695	<0.001
Pyridoxine (mcg)	−0.3828	−0.5970	−0.1687	<0.001
Vitamin B12 (mcg)	−0.2223	−0.2489	−0.1957	<0.001
Calcium (mg)	−0.0004	−0.0009	0.0001	0.127
Sodium (mg)	−0.0001	−0.0001	0.0002	0.507
Carbohydrate (g)	0.0049	0.0032	0.0065	<0.001
Vegetable protein (g)	0.1783	0.1681	0.1885	<0.001
Polyunsaturated fat	0.1221	0.0961	0.1481	<0.001
Fiber (g)	0.2080	0.1971	0.2189	<0.001
Vitamin A (RE)	0.0002	0.0001	0.0002	<0.001
Vitamin E (mg)	0.1466	0.1179	0.1752	<0.001
Vitamin K (mg)	0.0142	0.0131	0.0154	<0.001
Vitamin C (mg)	0.0075	0.0064	0.0086	<0.001
Thiamine (mg)	1.9870	1.7435	2.2305	<0.001
Folate (mcg)	0.0089	0.0082	0.0094	<0.001
Iron (mg)	0.2697	0.2381	0.3014	<0.001
Phosphorus (mg)	0.0012	0.0008	0.0015	<0.001
Potassium (mg)	0.0009	0.0008	0.0010	<0.001
Zinc (mg)	0.1079	0.0752	0.1401	<0.001
Selenium (mcg)	0.0192	0.0175	0.0208	<0.001
Magnesium (mg)	0.0188	0.0176	0.0199	<0.001
Copper (mg)	0.1235	0.0126	0.2344	0.029

^a^ Linear regression adjusted for sex and age.

**Table 4:** Characteristics of the individuals included in the study and their PHDI scores. ELSA-Brasil, 2008–2009.

	Individuals		PHDI Scores		
Characteristics	*n*	%	Mean	95% CI	*p*-Value *
Sex					0.101
Men	6723	45.5	60.2	59.9–60.5	
Women	8056	54.5	60.5	60.2–60.7	
Age group					<0.001
Adults	11,597	78.5	59.9	59.7–60.1	
Elderly	3182	21.5	61.8	61.4–62.3	
Smoking status					<0.001
Non-smokers	12,862	87.0	60.6	60.4–60.8	
Smokers	1916	13.0	59.0	58.5–59.5	
Physical activity level ^§^					<0.001
Low	11,218	77.0	59.8	59.6–60.0	
Moderate	2030	14.0	62.1	61.6–62.7	
Vigorous	1314	9.0	61.9	61.2–62.5	

* Student’s *t*-test or ANOVA. § *n* = 14.561.

**Table 5:** Association between Planetary Health Diet Index and overall dietary quality and carbon footprint. ELSA-Brasil, 2008–2009.

Regression Models	Bivariate			
	β	95% CI		*p*-Value
Model				
BHEI-R	0.4722	0.4506	0.4939	<0.001
Carbon footprint (kg/CO2eq/1000 kcal)	−1.3075	−1.5603	−1.0546	<0.001

Model adjusted for age and sex. BHEI-R: Brazilian Healthy Eating Index Revised.

## Supplementary Materials

The following are available online at https://www.mdpi.com/article/10.3390/nu13051698/s1. Table S1. Example of foods and ingredients included in the PHDI components. Figure S1. Normal distribution of the Planetary Health Diet Index. ELSA-Brasil, 2008–2009; Table S2. Correlations between Planetary Health Diet Index components and each component with total energy intake. ELSA-Brasil, 2008–2009; Table S3. Mean and 95% CI of the Brazilian Health Eating Index Revised (BHEI-R) and carbon footprint, grouped by sex and age group. ELSA-Brasil, 2008–2009.
