# Supplementary material for: Development and Validation of an Index Based on EAT-Lancet Recommendations: The Planetary Health Diet Index
Source: Nutrients. 2021 May 17;13(5):1698. doi: 10.3390/nu13051698 (PMC8156093; doi:10.3390/nu13051698)
Supplement: Supplementary file 1 [file nutrients-13-01698-s001.zip › nutrients-1187843-supplementary.pdf]

Table S1. Example of foods and ingredients included in the PHDI components.

Components	Example of included items
Nuts and peanuts	Nuts, pistachios, almonds, peanuts, and coconut pulp and milk
Legumes	Beans, pulses, lentils, chickpeas, peas, soybeans. Includes soy foods (e.g., soymilks, tofu, and textured soy protein)
Fruits	All fruits, including fruits used in juices, nectars and punches. Includes coconut water.
Vegetables	All vegetables, except tubers.
Whole cereals	Brown rice, whole bread, and oat flakes
Eggs	Chicken and other poultry eggs
Fish and seafood	Fish and seafood, such as mussels and shrimp
Tubers and potatoes	All types of potatoes and cassava as well as their derivatives flours
Dairy	Cow, goat and buffalo milks, yogurts, and cheeses
Vegetable oils	Vegetable oils used in recipes, such as soybean oil, sunflower oil and olive oil
Dark green vegetables (DGV)	Broccoli, arugula, cabbage, chicory, and watercress
Red and orange vegetables (ReV)	Tomatoes, beetroots, carrots, and pumpkins
Red meat	Beef, lamb and pork, including their processed meats (e.g., sausage, ham and salami)
Chicken and substitutes	Chicken and other poultry, including their processed meats (e.g.: pate). Includes intakes exceeding the upper limit of eggs and/or fish and seafood
Animal fats	Lard, tallow, butter and other dairy fats (e.g., sour cream and cheese cream)
Added sugars	All sweetener intake, including the table white or brown sugar and honey used as ingredients in processed or culinary products and the added sugar to manufactured foods and beverages

Figure S1. Normal distribution of the Planetary Health Diet Index. ELSA-Brasil, 2008-2009.

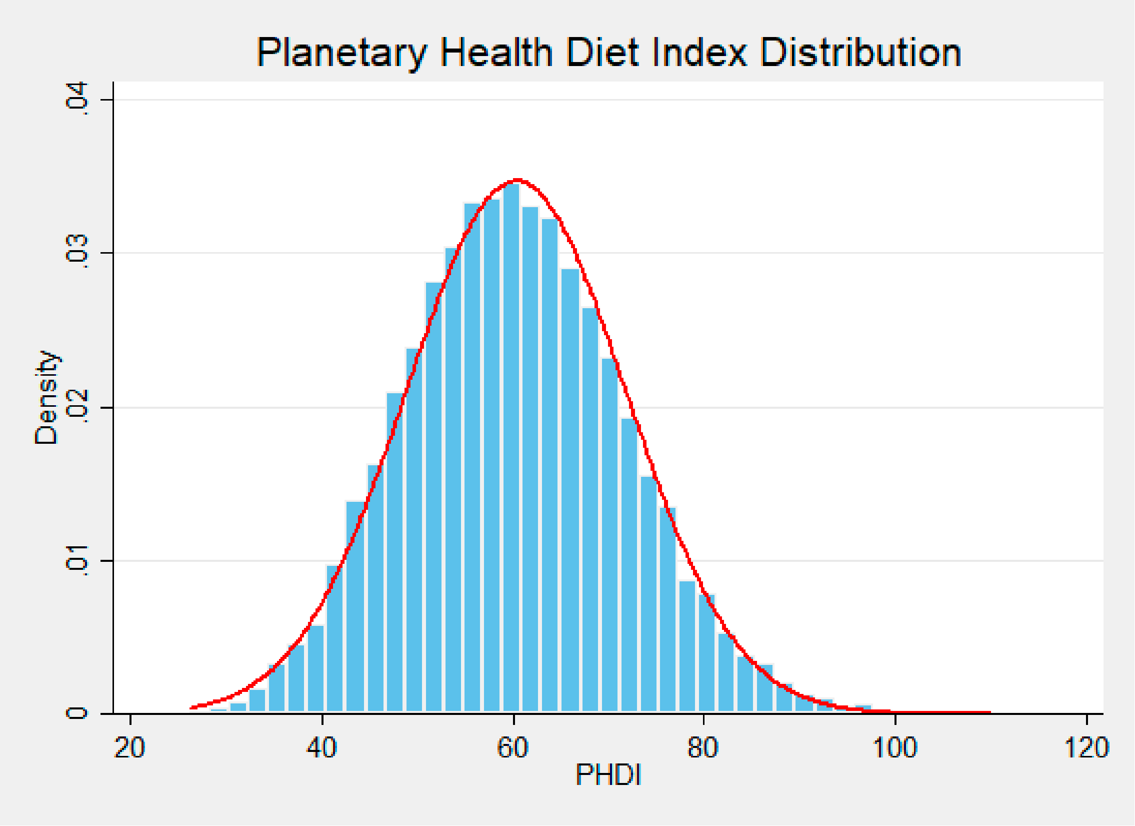

Table S2. Correlations between Planetary Health Diet Index components and each component with total energy intake. ELSA-Brasil, 2008-2009.

Component	Red meat	Nuts and peanuts	Legumes	Chicken and substitutes	Fish and seafood	Eggs	Fruits	Vegetables	DGV/total ratio	ReV/total ratio	Whole cereals	Tubers	Dairy	Unsaturated oils	Animal fats	Added sugars
Nuts and peanuts	0.12**															
Legumes	-0.01	-0.15**														
Chicken and substitutes	0.04**	0.08**	0.04**													
Fish and seafood	-0.09**	0.01	0.04**	**0.25****												
Eggs	-0.03**	0.03*	0.01	**0.19****	0.06**											
Fruits	0.03**	0.09**	-0.12**	0.01	0.04**	0.01										
Vegetables	-0.01	0.03*	0.00**	-0.02*	0.05**	-0.01	0.08**									
DGV/total ratio	0.06**	0.12**	-0.04**	0.01	-0.03*	0.00	0.08**	-0.03**								
ReV/total ratio	0.05**	0.10**	-0.07**	0.03*	-0.03*	0.01	0.08**	-0.17**	0.24**							
Whole cereals	0.18**	**0.19****	-0.12**	0.07**	-0.01	0.03**	0.08**	-0.00	0.15**	0.16**						
Tubers	0.02*	0.04**	-0.00	0.03**	-0.01	0.03**	0.00	-0.13**	**0.32****	**0.45****	0.08**					
Dairy	-0.03*	-0.02*	0.18**	-0.12**	-0.04**	-0.03**	-0.02*	0.04**	-0.06**	-0.13**	-0.06**	-0.09**				
Unsaturated oils	-0.10**	-0.19**	0.04**	-0.13**	-0.01	-0.04**	-0.16**	0.05**	-0.21**	-0.25**	-0.20**	-0.19**	0.16**			
Animal fats	0.05**	0.03*	0.08**	-0.03**	-0.07**	0.01	-0.01	-0.05**	0.10**	0.10**	0.09**	0.08**	-0.01	0.02		
Added sugars	0.02*	0.06**	0.04**	-0.06**	-0.08**	-0.02	0.03*	0.01	0.13**	0.09**	0.07**	0.04**	0.01	-0.09**	0.27**	
Energy	-0.10**	-0.07**	0.23**	0.00	0.11**	0.06**	-0.03*	0.03*	-0.20**	-0.21**	-0.18**	-0.10**	0.12**	0.14**	-0.09**	-0.16**

*p<0.05; **p<0.001

Table S3. Mean and 95% CI of the Brazilian Health Eating Index Revised (BHEI-R) and carbon footprint, grouped by sex and age group. ELSA-Brasil, 2008-2009.

	Individuals	BHEI-R			Carbon footprint[a]		
Characteristics	%	mean	95% CI	p-value*	mean	95% CI	p-value*
Gender				<0.001			0.008
Men	45.5	68.3	68.1 – 68.5		1.91	1.89 – 1.93	
Women	54.5	72.0	71.9 – 72.2		1.94	1.92 – 1.96	
Age group				<0.001			0.306
Adults	78.5	69.7	69.5 – 69.8		1.93	1.91 – 1.94	
Elderly	21.5	72.8	72.5 – 73.1		1.94	1.92 – 1.97	

*t test. [a]Carbon footprint: greenhouse gas emissions (kg CO2eq/1000 kcal per day).
